# Identification of genes with altered expression in male and female Schlager hypertensive mice

**DOI:** 10.1186/s12881-014-0101-x

**Published:** 2014-08-30

**Authors:** Christine L Chiu, Kristy L Jackson, Nerissa L Hearn, Nicole Steiner, Geoffrey A Head, Joanne M Lind

**Affiliations:** School of Medicine, University of Western Sydney, Locked Bag 1797, Penrith, NSW 2751 Australia; Neuropharmacology Laboratory, Baker IDI Heart and Diabetes Research Institute, Victoria, Australia; Department of Pharmacology, Monash University, Victoria, Australia

**Keywords:** Schlager mouse, Genome wide gene expression

## Abstract

**Background:**

Numerous studies have shown sex differences in the onset and severity of hypertension. Despite these sex-differences the majority of animal studies are carried out in males. This study investigated expression changes in both male and female hypertensive mouse kidneys to identify common mechanisms that may be involved in the development of hypertension.

**Methods:**

The Schlager hypertensive mouse model (BPH/2J) and its normotensive control (BPN/3J) were used in this study. Radiotelemetry was performed on 12 to 13 week old BPH/2J and BPN/3J male and female animals. Affymetrix GeneChip Mouse Gene 1.0 ST Arrays were performed in kidney tissue from 12 week old BPH/2J and BPN/3J male and female mice (n = 6/group). Genes that were differentially expressed in both male and female datasets were validated using qPCR.

**Results:**

Systolic arterial pressure and heart rate was significantly higher in BPH/2J mice compared with BPN/3J mice in both males and females. Microarray analysis identified 153 differentially expressed genes that were common between males and females (70 upregulated and 83 downregulated). We validated 15 genes by qPCR. Genes involved in sympathetic activity (*Hdc*, *Cndp2*), vascular ageing (*Edn3*), and telomere maintenance (*Mcm6*) were identified as being differentially expressed between BPH/2J and BPN/3J comparisons. Many of these genes also exhibited expression differences between males and females within a strain.

**Conclusions:**

This study utilised data from both male and female animals to identify a number of genes that may be involved in the development of hypertension. We show that female data can be used to refine candidate genes and pathways, as well as highlight potential mechanisms to explain the differences in prevalence and severity of disease between men and women.

**Electronic supplementary material:**

The online version of this article (doi:10.1186/s12881-014-0101-x) contains supplementary material, which is available to authorized users.

## Background

Essential hypertension is a leading cause of morbidity and mortality worldwide. Risk factors for the development of hypertension include a combination of environmental lifestyle factors as well as genetic factors. Significant sex differences in incidence and severity have long been recognized in the prevalence of hypertension [1]. These differences between males and females have suggested that the pathogenesis of human hypertension is modified by sex. The underlying mechanism that leads to disease may however be common to both sexes. The majority of studies utilising animal models of disease are carried out in male animals only, which does not allow for identification of common pathways in both sexes.

The Schlager hypertensive mouse model, (BPH/2J) and its normotensive control (BPN/3J) are a model of spontaneous hypertension that were derived via selective breeding for high and normal blood pressure. The BPH/2J strain mimics human disease with elevated blood pressure, increased heart rate, and early mortality. Previous studies have investigated gene expression changes in BPH/2J and BPN/3J male mice [2,3]. One study identified expression changes during early and established hypertension in the hypothalamus [2]. At both time points, genes involved in inflammation and oxidative stress were identified as being differentially expressed between BPH/2J and BPN/3J animals. Puig *et al.* compared gene expression in kidney, aorta, heart and liver tissue in male BPH/2J, BPN/3J and the BPL/1 J (low blood pressure) strains. They identified 222 transcripts that were differentially expressed across all tissues with expression changes in the same direction [3]. Whether genes and pathways with altered expression in male hypertensive mice are also altered in female mice, remains to be determined.

In the current study we performed gene expression arrays in kidney tissue from male and female BPH/2J and BPN/3J mice. We compared gene expression profiles between BPH/2J and BPN/3J strains, and identified genes that were common to both sexes. Focusing on biological processes common to both males and females will help to refine the mechanisms that are involved in the development of hypertension.

## Methods

### Blood pressure measurements

This study involved Schlager BPH/2J and BPN/3J mice. Previous radiotelemetry studies on 18 to 20 week old males have shown that blood pressure of BPH/2J hypertensive mice is consistently high and BPN/3J mice is consistently normal [4]. In this study, radiotelemetry was performed on 12 to 13 week old male (BPN/3J n = 8, BPH/2J n = 6) and female (BPN/3J n = 3, BPH/2J n = 4) mice as previously described [4,5]. In brief, blood pressure telemetry transmitters were implanted, and 10 days after surgery recordings of systolic arterial pressure were obtained. This had the Alfred Medical Research Education Precinct Animal Ethics Committee approval.

### Tissue collection

To avoid introducing gene expression changes due to stress handling from blood pressure measurement, blood pressure was not directly measured in the animals used for the microarrays. Kidney tissue was collected from 12 week old male and female hypertensive BPH/2J and age-matched normotensive BPN/3J mice (n = 6/group) during the inactive period, which corresponds to the trough in diurnal blood pressure. Mice were euthanized via cervical dislocation and tissues were snap frozen in liquid nitrogen and stored at -80°C. This study had University of Western Sydney Animal Ethics Committee approval.

### RNA extraction

Tissues were mechanically disrupted in the deep frozen state as previously described [6]. Total RNA was extracted from frozen tissue using the Qiagen RNeasy Mini Kit (Qiagen, Germany) according to the manufacturer’s recommendations, quantified using the NanoPhotometer (Implen, Germany) and stored at −80°C.

### Microarray analysis

RNA quality assessment and microarrays were performed by the Ramaciotti Centre for Gene Function Analysis, University of New South Wales, Australia. RNA quality was confirmed based on a RNA integrity number >8 using an electrophoresis bioanalyzer (2100 Agilent Bioanalyzer) and Affymetrix GeneChip Mouse Gene 1.0 ST Arrays were used. Microarray results were normalised using robust multiarray analysis (RMA). Differentially expressed genes between hypertensive and normotensive mice were identified using GenePattern 3.1 Comparative Marker Selection analysis. This analysis performs a two-sided t-test. Genes were selected based on a Bonferroni-corrected P value < 0.05. The data obtained has been deposited in the NCBI Gene Expression Omnibus (GEO) database according to the MIAME guidelines (accession number GSE54015).

### Gene ontology analysis

The GENECODIS (http://genecodis.cnb.csic.es) web-based software tool was used to perform gene ontology (GO) enrichment analysis [7,8]. The biological process and molecular functions were analysed simultaneously. Differentially expressed genes that were common in the male and female dataset were selected and were tested against the background set of all genes present in the Affymetrix GeneChip Mouse Gene 1.0 ST Array, using the default lowest GO annotation level. In addition, pathway enrichment analysis based on the PANTHER (protein annotation through evolutionary relationship) classification database (PANTHER pathway) was used to identify significant pathways.

### Real-time PCR

cDNA was synthesised using Bioscript™ Reverse Transcriptase and random hexamers (Bioline Pty Ltd, Australia) according to the manufacturer’s protocol. This was the same RNA used for the microarrays. Quantitative PCR (qPCR) was used to validate genes found to be differentially expressed on microarray. Beta Actin (bAct), glyceraldehyde 3-phosphate dehydrogenase (Gapdh), and hypoxanthine guanine phosphoribosyl transferase (Hprt1) were used as normalising genes (Additional file 1: Table S1). Individual reactions (10 μl) contained 2× Sensifast SYBR Mix (Bioline), forward and reverse primers (10 μM), and 4 μl of cDNA (or 4 μl water for no template controls). The PCR reactions were carried out in a MxPro3005P Real Time PCR System (Stratagene, Agilent Technologies, USA) followed by a dissociation curve. All samples were run in triplicate.

Cycle threshold (Ct) values for each sample were calculated using the MxPRo QPCR software (Stratagene, Agilent Technologies, USA). Triplicate Ct values were averaged and the quantity (Q) of each sample was calculated using the delta-delta Ct method. Q values from the normaliser genes were input into geNorm and the geometric means from these genes were used to generate a normalisation factor (NF) [9]. The Q values of the genes of interest were normalised by dividing by the NF value.

### Statistical analysis

Results are expressed as mean ± standard error, or mean fold changes in expression, and analysed using a general linear model. Statistical analysis was performed with PASW Statistics version 20. A P value of <0.05 was considered significant.

## Results

Radiotelemetry was performed on male and female BPH/2J and BPN/3J mice at 12 to 13 weeks of age. Male BPN/3J and BPH/2J and female BPH/2J exhibited significant differences in blood pressure and heart rate, between the day (inactive period) and night (active period), with blood pressure and heart rate increased during the night (active period). No differences in day and night blood pressure or heart rate were seen in female BPN/3J. In males, systolic arterial pressure, diastolic pressure and heart rate were significantly higher in BPH/2J compared with BPN/3J mice, regardless of time of day. In females, BPH/2J mice had significantly higher systolic arterial pressure and heart rate at both day and night when compared to BPN/3J. However, no significant difference in diastolic pressure was observed between BPH/2J and BPN/3J females (Table 1). Within the BPH/2J strain no significant difference in 24 hour systolic arterial pressure or diastolic pressure or heart rate was observed between males and females. In the BPN/3J strain, no difference in 24 hour systolic arterial pressure or diastolic pressure was observed, however females had a significantly higher 24 hour heart rate (p = 0.02) when compared to males.

**Table 1:** **Radiotelemetry measurements in 12 to 13 week old BPH/2J and BPN/3J mice**

	**BPN/3J (mean ± SE)**	**BPH/2J (mean ± SE)**	**p value**
***Male***	**n = 6**	**n = 8**	
Systolic BP - Day (mmHg)	105.7 ± 1.8	133.5 ± 2.9	<0.001
Systolic BP- Night (mmHg)	114.2 ± 1.4	154.4 ± 4.2	<0.001
Systolic BP - 24 hr (mmHg)	109.9 ± 0.8	143.9 ± 4.0	<0.001
Diastolic BP - Day (mmHg)	78.2 ± 2.0	100.3 ± 3.2	<0.001
Diastolic BP - Night (mmHg)	87.0 ± 0.7	116.5 ± 3.3	<0.001
Diastolic BP - 24 hr (mmHg)	82.6 ± 0.6	108.4 ± 1.3	<0.001
Heart rate - Day (beats/min)	358.7 ± 13.8	518.1 ± 26.3	0.001
Heart rate - Night (beats/min)	438.5 ± 16.3	627.3 ± 5.0	<0.001
Heart rate - 24 hr (beats/min)	398.6 ± 5.2	572.7 ± 25.2	<0.001
***Female***	**n = 3**	**n = 4**	
Systolic BP - Day (mmHg)	112.1 ± 1.4	128.5 ± 1.5	0.008
Systolic BP- Night (mmHg)	117.6 ± 3.8	148.3 ± 1.6	0.009
Systolic BP - 24 hr (mmHg)	115.4 ± 3.6	138.4 ± 1.2	0.009
Diastolic BP - Day (mmHg)	93.4 ± 2.3	93.5 ± 2.1	0.98
Diastolic BP - Night (mmHg)	97.3 ± 6.6	111.7 ± 1.8	0.17
Diastolic BP - 24 hr (mmHg)	95.3 ± 3.6	102.6 ± 0.8	0.36
Heart rate - Day (beats/min)	486.8 ± 15.5	551.7 ± 9.7	0.018
Heart rate - Night (beats/min)	505.8 ± 21.1	677.7 ± 7.4	0.001
Heart rate - 24 hr (beats/min)	496.3 ± 11.2	614.7 ± 7.3	0.022

Microarray expression analysis was performed on both male and female hypertensive (BPH/2J) and normotensive (BPN/3J) mice. Comparison between BPH/2J and BPN/3J was performed with male and female animals analysed separately. In males, 348 genes were found to be differentially expressed between BPH/2J and BPN/3J (Additional file 2: Table S2). Of these, 170 were upregulated and 178 were downregulated in the BPH/2J. In the females, 300 genes were found to be differentially expressed, with 152 upregulated and 150 downregulated in the BPH/2J (Additional file 3: Table S3). Of the genes that were identified as being differentially expressed, 70 upregulated and 83 downregulated genes were common between males and females.

Of the differentially expressed genes common in both the male and female lists, GO enrichment analysis was performed using GENECODIS [7,8] and biological processes and molecular functions were examined. Single categories that were significantly enriched in our gene set were transport (GO:0006810, p = 0.01), metabolic process (GO:0008152, p = 0.019) and hydrolase activity (GO:0016787, p = 0.038). In addition to these single GO terms, a significant set of genes were co-annotated with oxidation-reduction process and oxidoreductase activity (GO:0055114, GO:0016491, p = 0.026) or metabolic process and hydrolase activity (GO:0008152, GO:0016787, p = 0.025). Panther Pathway enrichment analysis was also performed and no pathways were found to be significantly represented in our gene list.

Genes were selected for validation by qPCR (Table 2). These genes were selected based on fold change differences, previous association with blood pressure regulation, and/or involvement in processes or pathways that may influence blood pressure. Of the genes that were differentially expressed in both the male and female, BPH/2J and BPN/3J comparisons, 15 out of 21 genes (71%) were validated on qPCR. The expression of these genes was also compared between males and females within a strain (Table 3). The expression of *Hdc*, *Edn3, Il10rb, Gabra3a*, *Kcnk1*, and *Serpinb8* was significantly higher in female kidneys compared to male kidneys in both the BPH/2J and BPN/3J strains. *Angplt7*, *Cndp2* and *Cyp4b1* expression was significantly lower in females when compared to males within a strain. The expression of *Pld1* and *Pak6* was significantly higher only in BPH/2J females, while the expression of *Vwa1* was significantly lower only in the BPN/3J females. No difference in expression between the sexes within a strain was observed for *Mcm6*, *Dna2* or *Pter*.

**Table 2:** **Genes common in males and females that were validated by qPCR**

	**Male**			**Female**		
**Gene**	**FC qPCR**	**P value qPCR**	**FC (array)**	**FC qPCR**	**P value qPCR**	**FC (array)**
*Hdc*	1154	<0.001	1.86	5.0	<0.001	1.10
*Gabra3*	97	<0.001	1.53	128	<0.001	1.56
*Angptl7*	53	<0.001	1.47	89	<0.001	1.68
*Cyp4b1*	12.2	<0.001	1.15	20.4	<0.001	1.39
*Edn3*	4.4	<0.001	1.14	3.8	<0.001	1.16
*Dna2*	1.7	0.001	1.12	1.4	0.02	1.08
*Cndp2*	1.4	0.01	1.12	1.9	0.03	1.08
*Mcm6*	−10	<0.001	−1.30	−8.7	<0.001	−1.30
*Vwa1*	−10	<0.001	−1.23	−3.0	<0.001	−1.14
*Kcnk1*	−3.3	<0.001	−1.15	−3.2	<0.001	−1.18
*Serpinb8*	−2.5	<0.001	−1.21	−2.7	<0.001	−1.22
*Pld1*	−2.0	0.001	−1.11	−1.4	0.02	−1.11
*Pter*	−1.7	0.001	−1.03	−1.5	<0.001	−1.03
*Pak6*	−1.7	<0.001	−1.10	−1.3	0.03	−1.11
*Il10rb*	−1.3	0.004	−1.07	−1.3	0.01	−1.06

Values represent mean fold changes (FC) between BPH/2J and BPN/3J samples relative to BPN/3J. Positive values indicate higher expression in BPH/2J samples while, negative values indicate lower expression in BPH/2J samples.

**Table 3:** **Quantitative PCR fold changes in females, relative to males within a strain**

	**BPH/2J**		**BPN/3J**	
**Gene**	**FC**	**P value**	**FC**	**P value**
*Hdc*	1.7	<0.001	388	<0.001
*Gabra3*	1.6	0.005	1.2	0.01
*Angptl7*	−2.8	<0.001	−4.7	<0.001
*Cyp4b1*	−13.2	<0.001	−22.2	<0.001
*Edn3*	1.7	0.005	1.9	0.005
*Dna2*	1.0	0.17	1.2	0.99
*Cndp2*	−1.6	<0.001	−2.1	0.002
*Mcm6*	1.9	0.03	1.7	0.03
*Vwa1*	1.8	0.01	−1.5	<0.001
*Kcnk1*	1.9	<0.001	2.1	<0.001
*Serpinb8*	1.8	<0.001	1.8	0.001
*Pld1*	1.9	0.001	1.4	0.03
*Pter*	1.2	0.12	1.2	0.08
*Pak6*	1.3	0.01	1.1	0.38
*Il10rb*	1.6	<0.001	1.7	<0.001

Values represent mean fold changes (FC) between male and female samples relative to males within a strain. Positive values indicate higher expression in female samples while, negative values indicate lower expression in female samples.

## Discussion

This study performed gene expression arrays in kidneys of BPH/2J and BPN/3J male and female mice. It is the first study to measure and compare gene expression profiles in female BPH/2J and BPN/3J mice to determine the mechanisms involved in the development of high blood pressure in these mice. The BPH/2J strain was generated via multiple generations of brother sister mating selecting for high blood pressure. Radiotelemetry in these mice found significantly increased systolic arterial pressure in both male and female hypertensive mice, compared with the normotensive strain, with no difference in blood pressure between males and females. This would suggest that a common mechanism is responsible for high blood pressure in both sexes. We found that while there were differences in the expression profiles between BPH/2J and BPN/3J depending on sex, approximately half of the differentially expressed genes were present in both male and female datasets. This allowed us to refine the list of possible candidate genes that were only differentially expressed in both sexes. This list included *Angptl7, Hdc*, *Cndp2*, *Dna2*, *Edn3*, which were up-regulated in the hypertensive mice and may have a physiological role in the regulation of high blood pressure. *Mcm6* and *Il10rb* were both down-regulated in the hypertensive mice and may also play a role high blood pressure. A previous study compared gene expression across multiple tissues between 12 week old male hypertensive and normotensive mice [3]. A number of genes, including *Angplt7*, *Hdc*, *Mcm6* and *Il10rb* that were identified as being differentially expressed in the kidneys of those animals were also identified in the kidneys of male and females in the current study.

We show that expression of this *Angptl7* is increased in BPH/2J kidneys, with expression being significantly higher in male kidneys compared to females within a strain. Angptl7 belongs to the angiopoietin like protein family. Several angiopoietin-like proteins have been shown to be involved in the regulation of angiogenesis as well as lipid and glucose metabolism [10]. Angptl7 is the least studied of all the angiopoietin-like proteins. Studies in patients with glaucoma, a condition characterised by increased intraocular pressure, have reported elevated *Angptl7* expression in the cornea [11]. Longitudinal cohort studies have also shown hypertension is associated with an elevated risk of developing glaucoma [12]. These studies, together with the increased expression of *Angptl7* observed in the kidneys of our hypertensive mice, suggests that *Angptl7* may be upregulated in response to increased vasculature pressure.

The BPH/2J has been shown to be a model of neurogenic hypertension and these mice have increased sympathetic activity [4]. Histidine decarboxylase (Hdc) and carnosine dipeptidase 2 (Cndp2) have been shown to be involved in sympathetic activity. These genes are part of the carosine- histidine - histamine metabolic pathway. Carnosine is broken down into histidine via the action of Cndp2, and then histidine is converted into histamine by Hdc. Carnosine is an antioxidant that has been reported to have a protective effect on kidneys as a scavenger of reactive oxygen species [13]. It has been shown to reduce renal sympathetic nerve activity in rats, and inhibit hypertension in DOCA salt hypertensive rats [14]. Additionally, a patient with congenital carnosinase deficiency, the inability to breakdown carnosine, was reported to have elevated levels of carnosine and hypotension [15]. *Cndp2* expression was significantly elevated in both BPH/2J males and females. Elevated levels of *Cndp2* may result in reduced carnosine, decreased protection against oxidative stress, and increased levels of histidine.

Hdc catalyses the conversion of histidine to histamine. Histamine is a neurotransmitter that is involved in the regulation of renal sympathetic activity and response to inflammation. In anesthetised rats a low dose of histamine suppressed renal sympathetic nerve activity and blood pressure, while a high dose of histamine elevated renal sympathetic nerve activity and blood pressure [16]. *Hdc* was previously reported to be significantly upregulated in the kidneys of 12 week male BPH/2J [3]. It is upregulated by estrogen and is expressed at higher levels in female kidneys compared to males [17]. This was reflected in our study, where the fold change between male BPH/2J and BPN/3J was significantly larger than the fold change between female BPH/2J and BPN/3J, due to the BPN/3J males having low *Hdc* expression. Increased expression of *Cndp2* and *Hdc* may result in higher levels of histamine in the BPH/2J animals which may contribute to the elevated sympathetic activity and blood pressure seen in this strain.

Genes that were involved in DNA replication, repair and telomere maintenance were identified in both male and female datasets. Mcm6 is a subunit of the Mcm2-7 complex, a replicative helicase that is involved in eukaryotic DNA replication. It is an essential component of the MCM complex as mutations in *Mcm6* result in the loss of helicase activity [18]. DNA2 is a nuclease/helicase that is essential for Okazaki fragment processing during DNA replication. Recent studies have also suggested a role for Mcm6 and Dna2 in telomere length maintenance [19–21]. As telomere length has been associated with hypertension [22], dysregulation in the expression of these genes may affect DNA replication and telomere maintenance may contribute the development of disease.

A number of the differentially expressed genes in both males and females were known to have links to blood pressure regulation. *Edn3* was identified as a candidate gene for hypertension in humans via a GWA study [23]. The expression of *Edn3* was found to be upregulated in BPH/2J animals. Females were also found to have significantly higher *Edn3* expression when compared to males within a strain. Increased expression of *Edn3* in the kidneys of ageing rats has suggested that it may be a marker for advanced ageing [24]. The prevalence of hypertension increases with increasing age [25]. This may suggest a role for premature ageing in hypertension.

Inflammation and oxidative stress are characteristic of hypertension. IL10 is an anti-inflammatory cytokine that can inhibit the synthesis of pro-inflammatory cytokines [26]. It functions by binding to its receptor IL10R and activates JAK and STAT pathways. DNA variants in *IL10rb*, a subunit of IL10R, have been associated with ischemic stroke in individuals with hypertension [27], and IL10 deficiency has been associated with preeclampsia, a condition characterised by high blood pressure during pregnancy [28,29]. The reduced expression of *IL10rb* seen in both male and female BPH/2J mice may result in reduced IL10 activity and increased inflammation in the hypertensive animals.

Many of the genes that were differentially expressed between BPH/2J and BPN/3J also exhibited expression differences between males and females within a strain. These differences suggests that while a common mechanism is most likely responsible for high blood pressure in the BPH/2J strain, differences in sex hormones will influence how gene/pathways respond to elevated blood pressure. Furthermore, these differences in expression may help to explain the differences in disease phenotype that are seen between men and women.

## Conclusion

This study has identified a combination of potential new candidate genes with no currently known links to hypertension, as well as genes that had been previously associated with hypertension and blood pressure control. The genes that we identified in both male and female BPH/2J hypertensive mice may be involved in the development or maintenance of hypertension. Our findings from the female dataset have helped to refine the possible pathways that are involved in the onset of hypertension in these animals, as well as highlight potential mechanisms that may explain the differences that are seen in the prevalence and severity of hypertension between men and women.

### Grants

CLC received a University of Western Sydney Early Career Seed Grant to undertake this project. JM Lind is supported by a National Health and Medical Research – Australian Biomedical Fellowship.

## Additional files

Additional file 1: Table S1 Primer sequences. Additional file 2: Table S2 Differentially expressed genes in the kidneys of 12 week old BPH/2J males, relative to BPN/3J males, using Bonferroni corrected p <0.05. Additional file 3: Table S3 Differentially expressed genes in the kidneys of 12 week old BPH/2J females, relative to BPN/3J females, using Bonferroni corrected p <0.05.
