# Supplementary material for: Identification of genes with altered expression in male and female Schlager hypertensive mice
Source: BMC Med Genet. 2014 Aug 30;15:101. doi: 10.1186/s12881-014-0101-x (PMC4355368; doi:10.1186/s12881-014-0101-x)
Supplement: Additional file 1: Table S1 — Primer sequences. [file 12881_2014_101_MOESM1_ESM.docx]

| **Primer** | **Primer Sequence 5’ to 3’** |
| --- | --- |
| *Hprt1* | **F:** TGACACTGGCAAAACAATGCA **R:** GGTCCTTTTCACCAGCAAGCT |
| *Gapdh* | **F:**GACTTCAACAGCAACTCCCAC **R:** TCCACCACCCTGTTGCTGTA |
| *bAct* | **F:** GCTGTATTCCCCTCCATCGTG **R:** CACGGTTGGCCTTAGGGTTCAG |
| *Hdc* | **F**: CTGGCTTGATTTCCCTTGTG **R:** AGTCCCTCACTGGCACAGAT |
| *Gabra3* | **F:** GAATCTTGGATCGGCTTTTG **R:** GCCAGAAGATTGTTCAGTGGA |
| *Angptl7* | **F:** GCACTGCCGAGAGCAAGTA **R:** CAGGAACTCGTCAGGAGGA |
| *Edn3* | **F:** GCCACCTGGACATCATCTG **R:** CAGTCTCCCGCATCTCTTCT |
| *Dna2* | **F:** CAGGGACAGGAAAGACAACC **R:** GATGGGCTAAAGACGCAATG |
| *Cndp2* | **F:** GCCGGAGAAGAGAGGAGAGA **R:** CCGTAAATGCACACGGTTTT |
| *Mcm6* | **F:** GCAGCTGTGGTGAGAGATGA **R:** TGCTGCAGCTAAAATGGATG |
| *Vwa1* | **F:** GCCGAGAAGCACCTACACTT **R:** TTGCCGCTAGGTACCAATTC |
| *Kcnk1* | **F:** GGGAAATTGGAATTGGGACT **R:** GTCAGGAAGAGGAGGGTGAA |
| *Serpinb8* | **F:** GCCCACTGACTAAGCTGGTT **R:** ATAGGGCAGAGCCAGAACCT |
| *Pld1* | **F:** CGCAGATATGAGCAACCTGA **R:** TTATGGGACAGCCAGAGAGG |
| *Pter* | **F:** AGCCTTGGTGGAGAATACGA **R:** CTCCACACTTGATGCTGGTG |
| *Pak6* | **F:** CTGCCAGCATCCATGTGTA **R:** CTGGAAGATGGGGATTCTG |
| *Il10rb* | **F:** GAGAGTCAGGGCTGAATTGG **R:** GTCCACGTCTCAGGCTCATT |
| *Cyp4b1* | **F:** CACCTGGACTTCCTCGACAT **R:** TCATCCCACTGGAAGGAGTC |
