# Supplementary material for: Identification of genes with altered expression in male and female Schlager hypertensive mice
Source: BMC Med Genet. 2014 Aug 30;15:101. doi: 10.1186/s12881-014-0101-x (PMC4355368; doi:10.1186/s12881-014-0101-x)
Supplement: Additional file 2: Table S2 — Differentially expressed genes in the kidneys of 12 week old BPH/2J males, relative to BPN/3J males, using Bonferroni corrected p <0.05. [file 12881_2014_101_MOESM2_ESM.docx]

**Table S2.** Differentially expressed genes in the kidneys of 12 week old BPH/2J males, relative to BPN/3J males, using Bonferroni corrected p <0.05

| **Gene** | **Description** | **Fold Change** | **p value** | |
| --- | --- | --- | --- | --- |
| 1300002K09Rik | RIKEN cDNA 1300002K09 gene | -1.16 | <0.001 |  |
| 1500011K16Rik | RIKEN cDNA 1500011K16 gene | -1.05 | 0.033 |  |
| 1810037I17Rik | RIKEN cDNA 1810037I17 gene | -1.30 | <0.001 |  |
| 3110007f17Rik | RIKEN cDNA 3110007F17 gene | 1.28 | 0.031 |  |
| 4930555G01Rik | RIKEN cDNA 4930555G01 gene | -1.13 | 0.010 |  |
| 4930579g22rik | RIKEN cDNA 4930579G22 gene | 1.10 | 0.038 |  |
| 4933409k07rik | RIKEN cDNA 4933409K07 gene | 1.12 | 0.027 |  |
| 5730407i07rik | RIKEN cDNA 5730407I07 gene | 1.54 | <0.001 |  |
| 5830417I10Rik | RIKEN cDNA 5830417I10 gene | -1.25 | 0.003 |  |
| A2m | alpha-2-macroglobulin | 1.20 | 0.027 |  |
| Aadac | arylacetamide deacetylase (esterase) | -1.07 | <0.001 |  |
| Ab041803 | cDNA sequence AB041803 | 1.26 | <0.001 |  |
| Abcc9 | ATP-binding cassette, sub-family C (CFTR/MRP), member 9 | 1.11 | 0.003 |  |
| Abhd14a | abhydrolase domain containing 14A | -1.13 | <0.001 |  |
| Abhd14b | abhydrolase domain containing 14b | -1.07 | <0.001 |  |
| Acadvl | acyl-Coenzyme A dehydrogenase, very long chain | 1.02 | 0.020 |  |
| Acox2 | acyl-Coenzyme A oxidase 2, branched chain | 1.15 | 0.000 |  |
| Acp5 | acid phosphatase 5, tartrate resistant | 1.07 | <0.001 |  |
| Acsm1 | acyl-CoA synthetase medium-chain family member 1 | -1.11 | 0.000 |  |
| Actr10 | ARP10 actin-related protein 10 homolog (S. cerevisiae) | 1.03 | 0.020 |  |
| Adam17 | a disintegrin and metallopeptidase domain 17 | 1.04 | 0.035 |  |
| Aga | aspartylglucosaminidase | -1.09 | <0.001 |  |
| Ahcy | S-adenosylhomocysteine hydrolase | -1.02 | 0.039 |  |
| Ahcyl2 | S-adenosylhomocysteine hydrolase-like 2 | 1.02 | 0.046 |  |
| Ak084462 | Mus musculus 13 days embryo heart cDNA, RIKEN full-length enriched library, clone | -1.22 | 0.049 |  |
| Ak5 | adenylate kinase 5 | 1.07 | 0.002 |  |
| Alad | aminolevulinate, delta-, dehydratase | 1.07 | 0.004 |  |
| Aldh1a7 | aldehyde dehydrogenase family 1, subfamily A7 | 1.40 | 0.010 |  |
| Aldh4a1 | aldehyde dehydrogenase 4 family, member A1 | 1.03 | <0.001 |  |
| Angptl2 | angiopoietin-like 2 | 1.03 | 0.013 |  |
| Angptl7 | angiopoietin-like 7 | 1.47 | 0.011 |  |
| Ankrd33b | ankyrin repeat domain 33B | 1.19 | 0.003 |  |
| Ankrd46 | ankyrin repeat domain 46 | -1.05 | 0.012 |  |
| Arfgef2 | ADP-ribosylation factor guanine nucleotide-exchange factor 2 | 1.05 | 0.018 |  |
| Arg2 | arginase type II | 1.12 | 0.046 |  |
| Arhgef19 | Rho guanine nucleotide exchange factor (GEF) 19 | 1.12 | 0.022 |  |
| Arhgef2 | rho/rac guanine nucleotide exchange factor (GEF) 2 | 1.04 | 0.025 |  |
| Arl16 | ADP-ribosylation factor-like 16 | 1.15 | 0.007 |  |
| Arl8a | ADP-ribosylation factor-like 8A | 1.02 | 0.022 |  |
| Armc8 |  | -1.03 | 0.033 |  |
| Asah2 | armadillo repeat containing 8 | 1.03 | 0.001 |  |
| Asns |  | -1.14 | 0.009 |  |
| Atg4a | N-acylsphingosine amidohydrolase 2 | -1.50 | 0.000 |  |
| Atp10d | asparagine synthetase | 1.28 | 0.000 |  |
| Atp6v1g1 | autophagy-related 4A (yeast) | 1.03 | 0.004 |  |
| Atp7b | ATPase, class V, type 10D | -1.14 | 0.013 |  |
| Bc040756 | ATPase, H+ transporting, lysosomal V1 subunit G1 | -1.08 | 0.044 |  |
| Bc067074 | ATPase, Cu++ transporting, beta polypeptide | 1.14 | <0.001 |  |
| Bdh2 | cDNA sequence BC040756 | -1.03 | 0.034 |  |
| Bik | cDNA sequence BC067074 | -1.19 | <0.001 |  |
| Bspry | 3-hydroxybutyrate dehydrogenase, type 2 | -1.08 | 0.030 |  |
| Bub1b | BCL2-interacting killer | -1.08 | 0.005 |  |
| C10orf11 | B-box and SPRY domain containing | -1.36 | 0.000 |  |
| C10orf125 | budding uninhibited by benzimidazoles 1 homolog, beta (S. cerevisiae) | -1.03 | 0.006 |  |
| C11orf67 | RIKEN cDNA 1700112E06 gene | 1.11 | <0.001 |  |
| C1orf192 | RIKEN cDNA 1810014F10 gene | -1.34 | 0.000 |  |
| C1orf198 | RIKEN cDNA 1810020D17 gene | 1.10 | 0.004 |  |
| C1orf56 | RIKEN cDNA 1700009P17 gene | 1.20 | 0.040 |  |
| C1orf91 | RIKEN cDNA 2310022B05 gene | -1.04 | 0.029 |  |
| C1ql3 | predicted gene 128 | 1.10 | 0.001 |  |
| C3orf25 | RIKEN cDNA 2510006D16 gene | 1.11 | <0.001 |  |
| C3orf58 | C1q-like 3 | -1.05 | 0.043 |  |
| C5orf28 | cDNA sequence BC060267 | -1.38 | 0.000 |  |
| C5orf34 | RIKEN cDNA 1190002N15 gene | -1.15 | 0.010 |  |
| C5orf62 | predicted gene 7120 | 1.09 | 0.007 |  |
| C6orf162 | RIKEN cDNA 4833420G17 gene | -1.03 | 0.034 |  |
| C6orf35 | RIKEN cDNA 2010002N04 gene | -1.08 | <0.001 |  |
| C7orf10 | RIKEN cDNA 1810030N24 gene | 1.06 | 0.003 |  |
| Cacnb4 | RIKEN cDNA 5730437N04 gene | -1.06 | 0.047 |  |
| Camta1 | RIKEN cDNA 5033411D12 gene | -1.15 | 0.022 |  |
| Cast | calcium channel, voltage-dependent, beta 4 subunit | 1.05 | <0.001 |  |
| Cbr2 | calmodulin binding transcription activator 1 | 1.38 | <0.001 |  |
| Cbx3 | calpastatin | 1.02 | 0.027 |  |
| Ccdc163 | carbonyl reductase 2 | -1.12 | 0.005 |  |
| Ccdc56 | chromobox homolog 3 (Drosophila HP1 gamma) | -1.03 | 0.001 |  |
| Ccl27a | coiled-coil domain containing 163 | -1.06 | 0.018 |  |
| Cd55 | coiled-coil domain containing 56 | 1.20 | 0.018 |  |
| Cd97 | chemokine (C-C motif) ligand 27A | -1.33 | 0.000 |  |
| Cdv3 | CD55 antigen | -1.02 | <0.001 |  |
| Ceacam21 | CD97 antigen | -1.10 | 0.019 |  |
| Clcn6 | carnitine deficiency-associated gene expressed in ventricle 3 | 1.06 | <0.001 |  |
| Clec12a | carcinoembryonic antigen-related cell adhesion molecule 2 | -1.11 | 0.016 |  |
| Clpx | chloride channel 6 | -1.05 | 0.021 |  |
| Cmas | C-type lectin domain family 12, member a | 1.04 | 0.002 |  |
| Cmbl | caseinolytic peptidase X (E.coli) | 1.05 | 0.022 |  |
| Cndp2 | cytidine monophospho-N-acetylneuraminic acid synthetase | 1.12 | 0.000 |  |
| Coasy | carboxymethylenebutenolidase-like (Pseudomonas) | -1.04 | <0.001 |  |
| Col6a6 | CNDP dipeptidase 2 (metallopeptidase M20 family) | 1.19 | 0.010 |  |
| Commd2 | Coenzyme A synthase | -1.12 | <0.001 |  |
| Cops8 | collagen, type VI, alpha 6 | 1.04 | 0.038 |  |
| Crym | COMM domain containing 2 | -1.20 | <0.001 |  |
| Ctnnbip1 | COP9 (constitutive photomorphogenic) homolog, subunit 8 (Arabidopsis thaliana) | -1.05 | 0.025 |  |
| Ctsl2 | crystallin, mu | -1.03 | 0.013 |  |
| Cybb | catenin beta interacting protein 1 | -1.06 | 0.035 |  |
| Cyp1b1 | cathepsin L | 1.04 | 0.009 |  |
| Cyp4a10 | cytochrome b-245, beta polypeptide | 1.10 | 0.011 |  |
| Cyp4a31 | cytochrome P450, family 1, subfamily b, polypeptide 1 | 1.13 | 0.001 |  |
| Cyp4b1 | cytochrome P450, family 4, subfamily a, polypeptide 32 | 1.15 | 0.000 |  |
| Cysltr2 | cytochrome P450, family 4, subfamily a, polypeptide 31 | 1.28 | 0.042 |  |
| D14ertd449e | cytochrome P450, family 4, subfamily b, polypeptide 1 | 1.06 | 0.001 |  |
| D830030k20rik | cysteinyl leukotriene receptor 2 | -1.14 | 0.004 |  |
| Dao | DNA segment, Chr 14, ERATO Doi 449, expressed | -1.04 | 0.002 |  |
| Dap | RIKEN cDNA D830030K20 gene | -1.08 | 0.001 |  |
| Dcbld2 | D-amino acid oxidase | 1.07 | 0.004 |  |
| Dci | death-associated protein | 1.05 | 0.048 |  |
| Dcn | discoidin, CUB and LCCL domain containing 2 | 1.03 | 0.014 |  |
| Ddx41 | dodecenoyl-Coenzyme A delta isomerase (3,2 trans-enoyl-Coenyme A isomerase) | -1.04 | 0.007 |  |
| Deaf1 | decorin | -1.04 | 0.028 |  |
| Defb1 | DEAD (Asp-Glu-Ala-Asp) box polypeptide 41 | 1.10 | <0.001 |  |
| Dhdh | deformed epidermal autoregulatory factor 1 (Drosophila) | -1.12 | 0.003 |  |
| Dhtkd1 | defensin beta 1 | 1.08 | 0.001 |  |
| Dis3l | dihydrodiol dehydrogenase (dimeric) | 1.05 | 0.008 |  |
| Dna2 | dehydrogenase E1 and transketolase domain containing 1 | 1.12 | <0.001 |  |
| Dnah9 | DIS3 mitotic control homolog (S. cerevisiae)-like | -1.07 | 0.032 |  |
| Dpys | DNA replication helicase 2 homolog (yeast) | 1.21 | 0.035 |  |
| Dynlt1a | dynein, axonemal, heavy chain 9 | 1.03 | <0.001 |  |
| Dynlt1c | dihydropyrimidinase | 1.03 | <0.001 |  |
| Echdc1 | dynein light chain Tctex-type 1A | 1.07 | <0.001 |  |
| Echdc3 | dynein light chain Tctex-type 1C | 1.06 | 0.040 |  |
| Edn3 | enoyl Coenzyme A hydratase domain containing 1 | 1.14 | 0.002 |  |
| Eif4a3 | enoyl Coenzyme A hydratase domain containing 3 | -1.27 | <0.001 |  |
| Enpep | endothelin 3 | -1.05 | 0.020 |  |
| Ensmusg00000068790 | eukaryotic translation initiation factor 4A3 | -1.17 | 0.007 |  |
| Ensmust00000082886 | glutamyl aminopeptidase | -1.60 | 0.000 |  |
| Ensmust00000093662 | predicted gene, ENSMUSG00000068790 | 1.12 | 0.002 |  |
| Ensmust00000112652 | ncrna | 1.95 | 0.000 |  |
| Ensmust00000124790 | ncrna | -1.05 | 0.050 |  |
| Ensmust00000149618 | cdna | -1.27 | <0.001 |  |
| Eps8 | cdna | -1.03 | 0.019 |  |
| Esm1 | cdna | 1.10 | 0.026 |  |
| Fahd2a | epidermal growth factor receptor pathway substrate 8 | -1.04 | 0.008 |  |
| Fam173a | endothelial cell-specific molecule 1 | 1.09 | 0.002 |  |
| Fam55c | fumarylacetoacetate hydrolase domain containing 2A | -1.22 | <0.001 |  |
| Fbln7 | family with sequence similarity 173, member A | 1.11 | 0.000 |  |
| Fbxo25 | family with sequence similarity 55, member C | 1.04 | 0.006 |  |
| Fbxo44 | fibulin 7 | -1.09 | 0.028 |  |
| Fcgr2b | F-box protein 25 | 1.08 | <0.001 |  |
| Fcgrt | F-box protein 44 | -1.06 | 0.005 |  |
| Fez2 | Fc receptor, IgG, low affinity IIb | 1.11 | <0.001 |  |
| Figf | Fc receptor, IgG, alpha chain transporter | 1.05 | 0.044 |  |
| Fmod | fasciculation and elongation protein zeta 2 (zygin II) | 1.16 | <0.001 |  |
| Ftcd | c-fos induced growth factor | -1.13 | 0.009 |  |
| Fuca1 | fibromodulin | -1.05 | <0.001 |  |
| Fv1 | formiminotransferase cyclodeaminase | 1.11 | <0.001 |  |
| G730007d18rik | fucosidase, alpha-L- 1, tissue | -1.27 | 0.006 |  |
| Gaa | Friend virus susceptibility 1 | -1.06 | 0.047 |  |
| Gabra3 | RIKEN cDNA G730007D18 gene | 1.53 | 0.000 |  |
| Gadd45gip1 | glucosidase, alpha, acid | -1.07 | <0.001 |  |
| Galc | gamma-aminobutyric acid (GABA) A receptor, subunit alpha 3 | -1.04 | <0.001 |  |
| Galnt3 | growth arrest and DNA-damage-inducible, gamma interacting protein 1 | -1.05 | 0.005 |  |
| Gamt | galactosylceramidase | 1.04 | 0.006 |  |
| Gart | UDP-N-acetyl-alpha-D-galactosamine | 1.03 | 0.017 |  |
| Gas5 | guanidinoacetate methyltransferase | -1.12 | <0.001 |  |
| Gas6 | phosphoribosylglycinamide formyltransferase | 1.18 | 0.000 |  |
| Gba | growth arrest specific 5 | -1.05 | <0.001 |  |
| Gdpd3 | growth arrest specific 6 | 1.29 | 0.000 |  |
| Genscan00000009258 | glucosidase, beta, acid | -1.15 | 0.002 |  |
| Gm10406 | glycerophosphodiester phosphodiesterase domain containing 3 | -1.17 | 0.003 |  |
| Gm10522 | cdna | 1.30 | 0.001 |  |
| Gm10524 | predicted gene 10406 | 1.18 | 0.002 |  |
| Gm10845 | predicted gene 10522 | -1.14 | 0.010 |  |
| Gm11711 | predicted gene 10524 | -1.18 | <0.001 |  |
| Gm13238 | predicted gene 10845 | -1.03 | <0.001 |  |
| Gm13306 | predicted gene 11711 | -1.06 | 0.034 |  |
| Gm14403 | predicted gene 13238 | -1.23 | <0.001 |  |
| Gm15348 | predicted gene 13306 | 1.16 | 0.010 |  |
| Gm16516 | predicted gene 14403 | -1.10 | 0.032 |  |
| Gm1943 | predicted gene 15348 | -1.08 | <0.001 |  |
| Gm1973 | predicted gene, Gm16516 | -1.11 | 0.012 |  |
| Gm2897 | WD repeat domain 70 pseudogene | -1.20 | 0.031 |  |
| Gm3002 | predicted gene 1973 | -1.21 | 0.004 |  |
| Gm3579 | predicted gene 2897 | -1.45 | 0.000 |  |
| Gm3696 | alpha-takusan pseudogene | -1.16 | 0.012 |  |
| Gm4983 | predicted gene 3579 | 1.12 | 0.033 |  |
| Gm5458 | predicted gene 3696 | -1.19 | 0.026 |  |
| Gm7455 | predicted gene 4983 | 1.19 | 0.009 |  |
| Gpr125 | predicted gene 5458 | 1.05 | 0.002 |  |
| Gramd1b | predicted gene 7455 | -1.03 | 0.010 |  |
| Grm7 | G protein-coupled receptor 125 | 1.19 | 0.019 |  |
| Gss | GRAM domain containing 1B | 1.02 | 0.027 |  |
| Gstk1 | glutamate receptor, metabotropic 7 | -1.05 | <0.001 |  |
| Gys2 | glutathione synthetase | 1.13 | <0.001 |  |
| H60b | glutathione S-transferase kappa 1 | 1.33 | 0.005 |  |
| Hdac1 | glycogen synthase 2 | 1.04 | 0.016 |  |
| Hdc | histocompatibility 60b | 1.86 | 0.000 |  |
| Hdhd3 | histone deacetylase 1 | 1.14 | 0.011 |  |
| Hebp1 | histidine decarboxylase | 1.12 | 0.049 |  |
| Hmgcs1 | haloacid dehalogenase-like hydrolase domain containing 3 | -1.06 | <0.001 |  |
| Hps5 | heme binding protein 1 | 1.13 | <0.001 |  |
| Hsd17b13 | 3-hydroxy-3-methylglutaryl-Coenzyme A synthase 1 | 1.23 | 0.017 |  |
| Hsd17b2 | Hermansky-Pudlak syndrome 5 homolog (human) | 1.11 | 0.013 |  |
| Hyal1 | hydroxysteroid (17-beta) dehydrogenase 13 | 1.13 | 0.035 |  |
| Iah1 | hydroxysteroid (17-beta) dehydrogenase 2 | 1.09 | <0.001 |  |
| Ica1 | hyaluronoglucosaminidase 1 | 1.11 | <0.001 |  |
| Idi1 | isoamyl acetate-hydrolyzing esterase 1 homolog (S. cerevisiae) | 1.07 | 0.008 |  |
| Il10rb | islet cell autoantigen 1 | -1.07 | 0.044 |  |
| Ino80 | isopentenyl-diphosphate delta isomerase | -1.03 | 0.044 |  |
| Itga1 | interleukin 10 receptor, beta | 1.03 | 0.008 |  |
| Ivd | INO80 homolog (S. cerevisiae) | -1.02 | <0.001 |  |
| Kcnk1 | integrin alpha 1 | -1.15 | <0.001 |  |
| Kctd2 | isovaleryl coenzyme A dehydrogenase | -1.07 | 0.001 |  |
| Kiaa0564 | potassium channel, subfamily K, member 1 | 1.02 | 0.014 |  |
| Kpna2 | potassium channel tetramerisation domain containing 2 | -1.06 | 0.045 |  |
| Krt10 | RIKEN cDNA 1300010F03 gene | -1.07 | 0.025 |  |
| Ldha | karyopherin (importin) alpha 2 | 1.04 | 0.002 |  |
| Leprot | keratin 10 | -1.07 | 0.002 |  |
| Leprotl1 | lactate dehydrogenase A | 1.05 | 0.003 |  |
| Lmbrd1 | leptin receptor overlapping transcript | 1.03 | 0.015 |  |
| Loc100293534 | leptin receptor overlapping transcript-like 1 | -1.17 | 0.002 |  |
| Loc280487 | LMBR1 domain containing 1 | -1.43 | <0.001 |  |
| Lphn1 | complement component 4A (Rodgers blood group) | -1.06 | 0.039 |  |
| Lppr1 | pol polyprotein | 1.24 | 0.043 |  |
| Lyplal1 | latrophilin 1 | 1.08 | 0.010 |  |
| Mas1 | RIKEN cDNA E130309F12 gene | -1.21 | 0.009 |  |
| Mcfd2 | lysophospholipase-like 1 | -1.08 | <0.001 |  |
| Mcm6 | MAS1 oncogene | -1.30 | <0.001 |  |
| Mfn2 | multiple coagulation factor deficiency 2 | -1.02 | 0.050 |  |
| Mgst3 | minichromosome maintenance deficient 6 | 1.11 | 0.005 |  |
| Mir692-1 | mitofusin 2 | 1.12 | 0.035 |  |
| Mme | microsomal glutathione S-transferase 3 | 1.10 | <0.001 |  |
| Mogat1 | microRNA 692-1 | 1.06 | 0.006 |  |
| Mpp7 | membrane metallo endopeptidase | -1.06 | 0.019 |  |
| Mrpl12 | monoacylglycerol O-acyltransferase 1 | -1.08 | 0.001 |  |
| Mrpl15 | membrane protein, palmitoylated 7 (MAGUK p55 subfamily member 7) | 1.05 | 0.041 |  |
| Ndufs2 | mitochondrial ribosomal protein L12 | 1.02 | 0.042 |  |
| Nell2 | mitochondrial ribosomal protein L15 | 1.30 | 0.007 |  |
| Nfe2l1 | NADH dehydrogenase (ubiquinone) Fe-S protein 2 | 1.03 | 0.046 |  |
| Nipa1 | NEL-like 2 (chicken) | -1.23 | 0.002 |  |
| Nipal2 | nuclear factor, erythroid derived 2,-like 1 | 1.22 | <0.001 |  |
| Nkiras1 | non imprinted in Prader-Willi/Angelman syndrome 1 homolog (human) | -1.07 | <0.001 |  |
| Nlrc4 | NIPA-like domain containing 2 | -1.42 | 0.002 |  |
| Nnt | NFKB inhibitor interacting Ras-like protein 1 | -1.03 | 0.030 |  |
| Nucb2 | NLR family, CARD domain containing 4 | -1.18 | <0.001 |  |
| Nusap1 | nicotinamide nucleotide transhydrogenase | -1.18 | 0.007 |  |
| Olfml2b | nucleobindin 2 | -1.10 | 0.007 |  |
| Oxgr1 | nucleolar and spindle associated protein 1 | 1.07 | 0.028 |  |
| P2rx4 | olfactomedin-like 2B | 1.03 | 0.011 |  |
| Padi2 | oxoglutarate (alpha-ketoglutarate) receptor 1 | -1.19 | <0.001 |  |
| Pak6 | purinergic receptor P2X, ligand-gated ion channel 4 | -1.10 | 0.001 |  |
| Parp8 | peptidyl arginine deiminase, type II | -1.04 | 0.047 |  |
| Pbld1 | p21 protein (Cdc42/Rac)-activated kinase 6 | 1.07 | 0.001 |  |
| Pcbp4 | poly (ADP-ribose) polymerase family, member 8 | 1.05 | <0.001 |  |
| Pdilt | phenazine biosynthesis-like protein domain containing | -1.10 | 0.005 |  |
| Pear1 | poly(rC) binding protein 4 | -1.08 | 0.032 |  |
| Pfn2 | protein disulfide isomerase-like, testis expressed | 1.04 | 0.003 |  |
| Pgm1 | platelet endothelial aggregation receptor 1 | 1.04 | 0.007 |  |
| Pigq | profilin 2 | -1.03 | 0.033 |  |
| Pigx | phosphoglucomutase 2 | -1.06 | <0.001 |  |
| Pik3c2g | phosphatidylinositol glycan anchor biosynthesis, class Q | -1.07 | 0.010 |  |
| Pla2g2c | phosphatidylinositol glycan anchor biosynthesis, class X | 1.17 | 0.023 |  |
| Plac9 | phosphatidylinositol 3-kinase, C2 domain containing, gamma polypeptide | 1.20 | <0.001 |  |
| Plcl2 | phospholipase A2, group IIC | -1.16 | 0.012 |  |
| Pld1 | placenta specific 9 | -1.11 | 0.018 |  |
| Plekhb1 | phospholipase C-like 2 | 1.27 | <0.001 |  |
| Plin2 | phospholipase D1 | -1.12 | 0.002 |  |
| Plod2 | pleckstrin homology domain containing, family B (evectins) member 1 | 1.06 | 0.026 |  |
| Pold3 | perilipin 2 | -1.06 | 0.022 |  |
| Polr1b | procollagen lysine, 2-oxoglutarate 5-dioxygenase 2 | -1.06 | 0.003 |  |
| Proc | polymerase (DNA-directed), delta 3, accessory subunit | -1.04 | <0.001 |  |
| Proz | polymerase (RNA) I polypeptide B | -1.09 | 0.008 |  |
| Psmd6 | protein C | 1.06 | 0.006 |  |
| Psph | protein Z, vitamin K-dependent plasma glycoprotein | 1.07 | 0.003 |  |
| Pter | proteasome (prosome, macropain) 26S subunit, non-ATPase, 6 | -1.03 | 0.007 |  |
| Ptpra | phosphoserine phosphatase | -1.04 | 0.001 |  |
| Ptprg | phosphotriesterase related | 1.06 | 0.007 |  |
| Pxmp2 | protein tyrosine phosphatase, receptor type, A | 1.08 | 0.013 |  |
| Qpctl | protein tyrosine phosphatase, receptor type, G | -1.05 | 0.014 |  |
| Rad51l3 | peroxisomal membrane protein 2 | 1.05 | 0.005 |  |
| Rbp7 | glutaminyl-peptide cyclotransferase-like | 1.18 | 0.002 |  |
| Rdm1 | RAD51-like 3 (S. cerevisiae) | 1.11 | 0.001 |  |
| Rfng | retinol binding protein 7, cellular | -1.08 | 0.011 |  |
| Rhbg | RAD52 motif 1 | 1.07 | <0.001 |  |
| Rhobtb2 | RFNG O-fucosylpeptide 3-beta-N-acetylglucosaminyltransferase | 1.07 | 0.021 |  |
| Rnaset2b | Rhesus blood group-associated B glycoprotein | 1.02 | 0.026 |  |
| Rnf13 | Rho-related BTB domain containing 2 | 1.07 | 0.005 |  |
| Rnf187 | ribonuclease T2B | -1.05 | 0.046 |  |
| Ropn1l | ring finger protein 13 | 1.10 | 0.010 |  |
| Rpl15 | ring finger protein 187 | -1.02 | 0.031 |  |
| Rpl35a | ropporin 1-like | -1.03 | 0.009 |  |
| Rplp1 | ribosomal protein L15 | 1.10 | 0.006 |  |
| Rtf1 | ribosomal protein L35A | 1.05 | 0.002 |  |
| Sbk1 | ribosomal protein, large, P1 | -1.11 | 0.049 |  |
| Scarna17 | Rtf1, Paf1/RNA polymerase II complex component, homolog (S. cerevisiae) | -1.04 | 0.022 |  |
| Scgb1c1 | SH3-binding kinase 1 | 1.26 | <0.001 |  |
| Scn1b | small Cajal body-specific RNA 17 | -1.10 | <0.001 |  |
| Sectm1 | secretoglobin, family 1C, member 1 | -1.50 | 0.002 |  |
| Sectm1b | sodium channel, voltage-gated, type I, beta | -1.22 | 0.000 |  |
| Sema3b | secreted and transmembrane 1A | 1.05 | 0.036 |  |
| Sema4g | secreted and transmembrane 1B | 1.07 | 0.040 |  |
| Serinc3 | sema domain, immunoglobulin domain (Ig), short basic domain, secreted, (semaphorin) 3B | 1.04 | <0.001 |  |
| Serpina1 | sema domain, immunoglobulin domain (Ig), transmembrane domain (TM) and short cytoplasmic domain, (semaphorin) 4G | 1.73 | 0.043 |  |
| Serpina3a | serine incorporator 3 | -1.27 | 0.031 |  |
| Serpinb8 | serine (or cysteine) peptidase inhibitor, clade A, member 1D | -1.21 | 0.002 |  |
| Sigmar1 | serine (or cysteine) peptidase inhibitor, clade A, member 3A | -1.04 | <0.001 |  |
| Slc12a2 | serine (or cysteine) peptidase inhibitor, clade B, member 8 | 1.07 | <0.001 |  |
| Slc12a7 | sigma non-opioid intracellular receptor 1 | -1.06 | 0.031 |  |
| Slc15a2 | solute carrier family 12, member 2 | 1.08 | <0.001 |  |
| Slc16a14 | solute carrier family 12, member 7 | -1.08 | 0.005 |  |
| Slc25a10 | solute carrier family 15 (H+/peptide transporter), member 2 | -1.06 | <0.001 |  |
| Slc26a1 | solute carrier family 16 (monocarboxylic acid transporters), member 14 | -1.06 | 0.014 |  |
| Slc35e1 | solute carrier family 25 (mitochondrial carrier, dicarboxylate transporter), member 10 | -1.04 | 0.010 |  |
| Slc47a1 | solute carrier family 26 (sulfate transporter), member 1 | -1.03 | 0.005 |  |
| Slc6a13 | solute carrier family 35, member E1 | -1.06 | 0.002 |  |
| Slc6a18 | solute carrier family 47, member 1 | -1.17 | <0.001 |  |
| Slco1a5 | solute carrier family 6 (neurotransmitter transporter, GABA), member 13 | 1.20 | 0.022 |  |
| Slco1a6 | solute carrier family 6 (neurotransmitter transporter), member 18 | 1.05 | 0.010 |  |
| Slit2 | solute carrier organic anion transporter family, member 1a4 | 1.06 | 0.013 |  |
| Snord53 | solute carrier organic anion transporter family, member 1a6 | 1.31 | <0.001 |  |
| Snx31 | slit homolog 2 (Drosophila) | 1.26 | <0.001 |  |
| Socs6 | small nucleolar RNA, C/D box 53 | -1.09 | <0.001 |  |
| Spink6 | sorting nexin 31 | -1.65 | <0.001 |  |
| Sppl2a | suppressor of cytokine signaling 6 | 1.03 | 0.047 |  |
| Ssr1 | serine peptidase inhibitor, Kazal type 6 | -1.04 | 0.002 |  |
| St7 | RIKEN cDNA 2010106G01 gene | -1.09 | 0.001 |  |
| Tex15 | signal sequence receptor, alpha | 1.22 | 0.001 |  |
| Tgm7 | suppression of tumorigenicity 7 | -1.23 | <0.001 |  |
| Thbs4 | testis expressed gene 15 | 1.12 | 0.020 |  |
| Tmc4 | transglutaminase 7 | 1.07 | 0.003 |  |
| Tmco1 | thrombospondin 4 | 1.04 | <0.001 |  |
| Tmem125 | transmembrane channel-like gene family 4 | -1.09 | 0.008 |  |
| Tmem144 | transmembrane and coiled-coil domains 1 | -1.08 | <0.001 |  |
| Tmem205 | transmembrane protein 125 | 1.05 | 0.001 |  |
| Tmem33 | transmembrane protein 144 | 1.03 | 0.048 |  |
| Tmem45b | transmembrane protein 205 | -1.10 | 0.012 |  |
| Tmem8a | transmembrane protein 33 | -1.06 | 0.001 |  |
| Tmprss9 | transmembrane protein 45b | -1.18 | 0.002 |  |
| Tomm20l | transmembrane protein 8 (five membrane-spanning domains) | -1.11 | 0.041 |  |
| Trappc2l | transmembrane protease, serine 9 | -1.08 | <0.001 |  |
| Trim13 | translocase of outer mitochondrial membrane 20 homolog (yeast)-like | -1.06 | 0.043 |  |
| Trmt12 | trafficking protein particle complex 2-like | -1.08 | 0.024 |  |
| Tstd2 | tripartite motif-containing 13 | -1.19 | 0.001 |  |
| Ttc38 | tRNA methyltranferase 12 homolog | -1.07 | <0.001 |  |
| Ttc8 | thiosulfate sulfurtransferase (rhodanese)-like domain containing 2 | 1.09 | 0.001 |  |
| Uevld | tetratricopeptide repeat domain 38 | 1.16 | <0.001 |  |
| Ugt1a7 | tetratricopeptide repeat domain 8 | -1.06 | <0.001 |  |
| Uqcc | UEV and lactate/malate dehyrogenase domains | -1.05 | 0.005 |  |
| Usp53 | UDP glucuronosyltransferase 1 family, polypeptide A9 | 1.06 | <0.001 |  |
| Vmn2r29 | ubiquinol-cytochrome c reductase complex chaperone, CBP3 homolog (yeast) | -1.11 | 0.009 |  |
| Vwa1 | ubiquitin specific peptidase 53 | -1.23 | 0.007 |  |
| Wtip | vomeronasal 2, receptor 29 | -1.07 | 0.009 |  |
| Ykt6 | von Willebrand factor A domain containing 1 | -1.05 | 0.017 |  |
| Zak | WT1-interacting protein | 1.07 | 0.001 |  |
| Zfp106 | YKT6 homolog (S. Cerevisiae) | 1.05 | 0.003 |  |
| Zfp125 | RIKEN cDNA B230120H23 gene | -1.55 | <0.001 |  |
| Zfp185 | zinc finger protein 106 | -1.06 | 0.030 |  |
| Znf605 | zinc finger protein 125 | 1.38 | <0.001 |  |
| Znf830 | zinc finger protein 185 | 1.23 | 0.002 |  |
| Zpld1 | zinc finger protein 605 | 1.23 | <0.001 |  |
