# Supplementary material for: Identification of genes with altered expression in male and female Schlager hypertensive mice
Source: BMC Med Genet. 2014 Aug 30;15:101. doi: 10.1186/s12881-014-0101-x (PMC4355368; doi:10.1186/s12881-014-0101-x)
Supplement: Additional file 3: Table S3 — Differentially expressed genes in the kidneys of 12 week old BPH/2J females, relative to BPN/3J females, using Bonferroni corrected p <0.05. [file 12881_2014_101_MOESM3_ESM.docx]

**Table S3.** Differentially expressed genes in the kidneys of 12 week old BPH/2J females, relative to BPN/3J females, using Bonferroni corrected p <0.05

| **Gene** | **Description** | **Fold Change** | **p value** |
| --- | --- | --- | --- |
| 0610010B08Rik | RIKEN cDNA 0610010B08 gene | 1.10 | 0.003 |
| 1110017F19Rik | RIKEN cDNA 1110017F19 gene | -1.18 | 0.032 |
| 1700049E17Rik1 | RIKEN cDNA 1700049E17 gene, gene 1 | -1.26 | <0.001 |
| 1810037I17Rik | RIKEN cDNA 1810037I17 gene | -1.32 | 0.027 |
| 2310001H12Rik | RIKEN cDNA 2310001H12 gene | -1.06 | 0.024 |
| 4930466F19Rik | RIKEN cDNA 4930466F19 gene | 1.27 | <0.001 |
| 4930503E14Rik | RIKEN cDNA 4930503E14 gene | -1.15 | <0.001 |
| 4933409K07Rik | RIKEN cDNA 4933409K07 gene | 1.12 | 0.047 |
| 5330426P16Rik | RIKEN cDNA 5330426P16 gene | 1.13 | <0.001 |
| 5730407I07Rik | RIKEN cDNA 5730407I07 gene | 1.47 | <0.001 |
| Aadac | arylacetamide deacetylase (esterase) | -1.07 | <0.001 |
| Abhd1 | abhydrolase domain containing 1 | -1.02 | 0.017 |
| Abhd14a | abhydrolase domain containing 14A | -1.14 | 0.002 |
| Abhd14b | abhydrolase domain containing 14b | -1.08 | 0.001 |
| Acp5 | acid phosphatase 5, tartrate resistant | 1.06 | 0.007 |
| Adra2b | adrenergic receptor, alpha 2b | -1.07 | 0.001 |
| Aga | aspartylglucosaminidase | -1.07 | 0.022 |
| Ahcyl2 | S-adenosylhomocysteine hydrolase-like 2 | 1.04 | 0.047 |
| Ak084462 | Mus musculus 13 days embryo heart cDNA, RIKEN full-length enriched library, clone | -1.23 | 0.001 |
| Alad | aminolevulinate, delta-, dehydratase | 1.06 | <0.001 |
| Angptl7 | angiopoietin-like 7 | 1.68 | <0.001 |
| Ankrd33b | ankyrin repeat domain 33B | 1.18 | <0.001 |
| Ap3m1 | adaptor-related protein complex 3, mu 1 subunit | -1.01 | 0.032 |
| Arfgef2 | ADP-ribosylation factor guanine nucleotide-exchange factor 2 | 1.04 | 0.002 |
| Arhgef2 | rho/rac guanine nucleotide exchange factor (GEF) 2 | 1.04 | 0.003 |
| Armcx1 | armadillo repeat containing, X-linked 1 | 1.09 | 0.001 |
| Armcx3 | armadillo repeat containing, X-linked 3 | 1.05 | 0.034 |
| Atg4a | autophagy-related 4A (yeast) | -1.43 | <0.001 |
| Atp10d | ATPase, class V, type 10D | 1.25 | <0.001 |
| Atp4a | ATPase, H+/K+ exchanging, gastric, alpha polypeptide | 1.31 | <0.001 |
| Atp6v1c1 | ATPase, H+ transporting, lysosomal V1 subunit C1 | -1.04 | 0.046 |
| Atp6v1f | ATPase, H+ transporting, lysosomal V1 subunit F | 1.01 | 0.018 |
| Bc040756 | cDNA sequence BC040756 | -1.05 | 0.038 |
| Bc067074 | cDNA sequence BC067074 | 1.07 | 0.004 |
| Bc126957 | Mus musculus cDNA clone IMAGE | 1.18 | 0.046 |
| Bid | BH3 interacting domain death agonist | -1.05 | 0.027 |
| Bik | BCL2-interacting killer | -1.18 | 0.003 |
| C10orf11 | RIKEN cDNA 1700112E06 gene | -1.32 | <0.001 |
| C14orf138 | predicted gene 71 | 1.05 | 0.018 |
| C14orf149 | RIKEN cDNA 2810055F11 gene | -1.07 | <0.001 |
| C16orf62 | RIKEN cDNA 9030624J02 gene | 1.05 | 0.004 |
| C1orf192 | RIKEN cDNA 1700009P17 gene | -1.29 | 0.001 |
| C1orf91 | RIKEN cDNA 2510006D16 gene | -1.02 | 0.032 |
| C20orf118 | predicted gene 1332 | 1.13 | 0.041 |
| C3orf25 | cDNA sequence BC060267 | 1.08 | 0.024 |
| C5orf28 | predicted gene 7120 | -1.31 | <0.001 |
| C5orf34 | RIKEN cDNA 4833420G17 gene | -1.18 | <0.001 |
| C6orf162 | RIKEN cDNA 1810030N24 gene | -1.03 | 0.040 |
| C6orf35 | RIKEN cDNA 5730437N04 gene | -1.06 | <0.001 |
| C7orf10 | RIKEN cDNA 5033411D12 gene | 1.06 | <0.001 |
| Cast | calpastatin | 1.03 | 0.014 |
| Cbr2 | carbonyl reductase 2 | 1.28 | 0.007 |
| Ccbl2 | cysteine conjugate-beta lyase 2 | -1.05 | 0.002 |
| Ccdc163 | coiled-coil domain containing 163 | -1.16 | 0.002 |
| Ccdc56 | coiled-coil domain containing 56 | -1.04 | <0.001 |
| Cd97 | CD97 antigen | -1.28 | 0.001 |
| Cda | cytidine deaminase | -1.03 | 0.023 |
| Cdh20 | cadherin 20 | 1.24 | 0.010 |
| Cdh4 | cadherin 4 | -1.09 | <0.001 |
| Cep97 | centrosomal protein 97 | -1.05 | 0.018 |
| Chrm3 | cholinergic receptor, muscarinic 3, cardiac | 1.07 | 0.004 |
| Cited1 | Cbp/p300-interacting transactivator with Glu/Asp-rich carboxy-terminal domain 1 | 1.21 | 0.005 |
| Cldn12 | claudin 12 | -1.05 | 0.006 |
| Clip4 | CAP-GLY domain containing linker protein family, member 4 | 1.16 | 0.001 |
| Cmtm6 | CKLF-like MARVEL transmembrane domain containing 6 | -1.04 | 0.031 |
| Cmtm7 | CKLF-like MARVEL transmembrane domain containing 7 | 1.11 | 0.046 |
| Cndp1 | carnosine dipeptidase 1 (metallopeptidase M20 family) | 1.14 | 0.005 |
| Cndp2 | CNDP dipeptidase 2 (metallopeptidase M20 family) | 1.08 | 0.002 |
| Commd2 | COMM domain containing 2 | -1.13 | 0.005 |
| Cox6b2 | cytochrome c oxidase subunit VIb polypeptide 2 | 1.09 | 0.047 |
| Cox7a1 | cytochrome c oxidase, subunit VIIa 1 | 1.05 | 0.019 |
| Crym | crystallin, mu | -1.17 | <0.001 |
| Ctsl2 | cathepsin L | -1.03 | 0.050 |
| Cwh43 | cell wall biogenesis 43 C-terminal homolog (S. cerevisiae) | -1.08 | 0.023 |
| Cyp4b1 | cytochrome P450, family 4, subfamily b, polypeptide 1 | 1.39 | <0.001 |
| Cysltr2 | cysteinyl leukotriene receptor 2 | 1.36 | 0.006 |
| D14ertd449e | DNA segment, Chr 14, ERATO Doi 449, expressed | 1.08 | <0.001 |
| Dao | D-amino acid oxidase | -1.05 | 0.002 |
| Dap | death-associated protein | -1.08 | 0.004 |
| Dcbld2 | discoidin, CUB and LCCL domain containing 2 | 1.07 | 0.017 |
| Ddc | dopa decarboxylase | -1.07 | 0.003 |
| Ddhd1 | DDHD domain containing 1 | 1.05 | 0.008 |
| Defb1 | defensin beta 1 | 1.09 | <0.001 |
| Dhtkd1 | dehydrogenase E1 and transketolase domain containing 1 | 1.09 | <0.001 |
| Dio1 | deiodinase, iodothyronine, type I | -1.12 | 0.047 |
| Dnajc28 | DnaJ (Hsp40) homolog, subfamily C, member 28 | -1.18 | 0.043 |
| Dna2 | DNA-replication helicase 2 homolog (yeast) | 1.08 | 0.043 |
| Dynlt1a | dynein light chain Tctex-type 1A | 1.03 | <0.001 |
| Dynlt1c | dynein light chain Tctex-type 1C | 1.03 | <0.001 |
| E330013p04rik | RIKEN cDNA E330013P04 gene | 1.26 | 0.007 |
| Edn3 | endothelin 3 | 1.16 | 0.028 |
| Efemp2 | epidermal growth factor-containing fibulin-like extracellular matrix protein 2 | 1.06 | 0.043 |
| Eif4a3 | eukaryotic translation initiation factor 4A3 | -1.27 | <0.001 |
| Ensmusg00000068790 | predicted gene, ENSMUSG00000068790 | -1.19 | <0.001 |
| Ensmust00000082886 | ncrna | -1.52 | 0.025 |
| Ensmust00000082910 | ncrna | 1.07 | 0.011 |
| Ensmust00000124790 | cdna | -1.03 | <0.001 |
| Ensmust00000137619 | cdna | -1.07 | <0.001 |
| Ensmust00000147499 | cdna | 1.10 | 0.006 |
| Entpd4 | ectonucleoside triphosphate diphosphohydrolase 4 | -1.03 | 0.020 |
| Esrp1 | epithelial splicing regulatory protein 1 | -1.11 | 0.004 |
| Fam132a | family with sequence similarity 132, member A | -1.03 | 0.016 |
| Fam173a | family with sequence similarity 173, member A | 1.09 | 0.001 |
| Fbxo44 | F-box protein 44 | -1.07 | 0.028 |
| Fez2 | fasciculation and elongation protein zeta 2 (zygin II) | 1.10 | <0.001 |
| Fgf1 | fibroblast growth factor 1 | -1.07 | <0.001 |
| Fggy | FGGY carbohydrate kinase domain containing | -1.04 | 0.047 |
| Fuca1 | fucosidase, alpha-L- 1, tissue | -1.05 | 0.011 |
| Gabra3 | gamma-aminobutyric acid (GABA) A receptor, subunit alpha 3 | 1.56 | 0.006 |
| Gadd45gip1 | growth arrest and DNA-damage-inducible, gamma interacting protein 1 | -1.08 | <0.001 |
| Gart | phosphoribosylglycinamide formyltransferase | 1.03 | 0.035 |
| Gas2 | growth arrest specific 2 | -1.03 | 0.049 |
| Gas5 | growth arrest specific 5 | -1.13 | <0.001 |
| Gas6 | growth arrest specific 6 | 1.17 | 0.002 |
| Gba | glucosidase, beta, acid | -1.07 | 0.003 |
| Gdpd3 | glycerophosphodiester phosphodiesterase domain containing 3 | 1.18 | <0.001 |
| Gfra2 | glial cell line derived neurotrophic factor family receptor alpha 2 | -1.17 | 0.017 |
| Gjb4 | gap junction protein, beta 4 | -1.20 | 0.046 |
| Glt25d2 | glycosyltransferase 25 domain containing 2 | 1.10 | 0.029 |
| Gm10406 | predicted gene 10406 | -1.21 | <0.001 |
| Gm10524 | predicted gene 10524 | 1.19 | 0.003 |
| Gm10732 | predicted gene 10732 | 1.29 | 0.016 |
| Gm10845 | predicted gene 10845 | -1.16 | 0.034 |
| Gm11428 | predicted gene 11428 | -1.41 | 0.010 |
| Gm13238 | predicted gene 13238 | -1.03 | 0.003 |
| Gm13306 | predicted gene 13306 | -1.07 | 0.004 |
| Gm14305 | predicted gene, 100043387 | 1.10 | 0.003 |
| Gm14326 | predicted gene 14326 | -1.02 | 0.039 |
| Gm14403 | predicted gene 14403 | -1.24 | <0.001 |
| Gm14430 | predicted gene 14430 | 1.10 | 0.003 |
| Gm15772 | ribosomal protein L26 pseudogene | -1.05 | 0.007 |
| Gm1943 | WD repeat domain 70 pseudogene | -1.08 | <0.001 |
| Gm1973 | predicted gene 1973 | -1.21 | 0.009 |
| Gm2897 | predicted gene 2897 | -1.27 | <0.001 |
| Gm3002 | alpha-takusan pseudogene | -1.22 | <0.001 |
| Gm3696 | predicted gene 3696 | -1.20 | 0.002 |
| Gm3893 | predicted gene 3893 | 1.13 | 0.012 |
| Gm5458 | predicted gene 5458 | -1.16 | 0.028 |
| Gm5622 | predicted gene 5622 | -1.12 | 0.018 |
| Gm773 | predicted gene 773 | 1.25 | 0.026 |
| Gpr125 | G protein-coupled receptor 125 | 1.04 | <0.001 |
| Gprc5a | G protein-coupled receptor, family C, group 5, member A | 1.06 | 0.016 |
| Gprc5b | G protein-coupled receptor, family C, group 5, member B | 1.09 | 0.023 |
| Grm7 | glutamate receptor, metabotropic 7 | 1.22 | 0.034 |
| Gsto1 | glutathione S-transferase omega 1 | 1.05 | 0.005 |
| H60b | histocompatibility 60b | 1.21 | <0.001 |
| Hdc | histidine decarboxylase | 1.10 | 0.046 |
| Hdhd3 | haloacid dehalogenase-like hydrolase domain containing 3 | 1.13 | 0.007 |
| Hgd | homogentisate 1, 2-dioxygenase | -1.02 | 0.044 |
| Hmgcs1 | 3-hydroxy-3-methylglutaryl-Coenzyme A synthase 1 | -1.07 | 0.001 |
| Hoxa10 | homeobox A10 | 1.04 | 0.041 |
| Hps5 | Hermansky-Pudlak syndrome 5 homolog (human) | 1.11 | 0.002 |
| Hrsp12 | heat-responsive protein 12 | -1.02 | 0.012 |
| Hsd17b14 | hydroxysteroid (17-beta) dehydrogenase 14 | 1.19 | 0.049 |
| Iah1 | isoamyl acetate-hydrolyzing esterase 1 homolog (S. cerevisiae) | 1.09 | <0.001 |
| Ifi35 | interferon-induced protein 35 | 1.10 | 0.010 |
| Il10rb | interleukin 10 receptor, beta | -1.06 | 0.006 |
| Inpp5b | inositol polyphosphate-5-phosphatase B | -1.06 | 0.029 |
| Itga1 | integrin alpha 1 | 1.03 | 0.011 |
| Ivd | isovaleryl coenzyme A dehydrogenase | -1.04 | 0.019 |
| Kcnk1 | potassium channel, subfamily K, member 1 | -1.18 | <0.001 |
| Kctd2 | potassium channel tetramerisation domain containing 2 | -1.05 | 0.011 |
| Kidins220 | kinase D-interacting substrate 220 | 1.03 | 0.040 |
| Klhdc1 | kelch domain containing 1 | 1.09 | 0.027 |
| Klk1 | kallikrein 1 | 1.02 | 0.002 |
| Laptm4b | lysosomal-associated protein transmembrane 4B | 1.04 | 0.001 |
| Ldha | lactate dehydrogenase A | 1.04 | <0.001 |
| Leprot | leptin receptor overlapping transcript | -1.07 | 0.030 |
| Leprotl1 | leptin receptor overlapping transcript-like 1 | 1.06 | 0.001 |
| Lig3 | ligase III, DNA, ATP-dependent | 1.05 | <0.001 |
| Loc100043371 | similar to development and differentiation enhancing factor 2 | 1.04 | 0.006 |
| Loc280487 | pol polyprotein | -1.34 | <0.001 |
| Loc388630 | predicted gene 12824 | -1.07 | <0.001 |
| Loxl2 | lysyl oxidase-like 2 | 1.11 | 0.003 |
| Lyrm7 | LYR motif containing 7 | -1.14 | 0.004 |
| Lzts2 | leucine zipper, putative tumor suppressor 2 | 1.05 | 0.010 |
| Mcfd2 | multiple coagulation factor deficiency 2 | -1.07 | 0.002 |
| Mcm6 | minichromosome maintenance deficient 6 (MIS5 homolog, S. pombe) (S. cerevisiae) | -1.30 | 0.007 |
| Mogat1 | monoacylglycerol O-acyltransferase 1 | 1.17 | <0.001 |
| Mogat2 | monoacylglycerol O-acyltransferase 2 | 1.15 | 0.001 |
| Mpp7 | membrane protein, palmitoylated 7 (MAGUK p55 subfamily member 7) | -1.07 | 0.028 |
| Mro | maestro | -1.17 | 0.013 |
| Mrpl12 | mitochondrial ribosomal protein L12 | -1.07 | <0.001 |
| Mrpl15 | mitochondrial ribosomal protein L15 | 1.05 | 0.010 |
| Mto1 | mitochondrial translation optimization 1 homolog (S. cerevisiae) | -1.03 | 0.003 |
| Naaladl1 | N-acetylated alpha-linked acidic dipeptidase-like 1 | 1.16 | 0.004 |
| Ndufs2 | NADH dehydrogenase (ubiquinone) Fe-S protein 2 | 1.03 | 0.016 |
| Nell2 | NEL-like 2 (chicken) | 1.31 | <0.001 |
| Nipal2 | NIPA-like domain containing 2 | 1.22 | <0.001 |
| Nkiras1 | NFKB inhibitor interacting Ras-like protein 1 | -1.07 | 0.036 |
| Nnt | nicotinamide nucleotide transhydrogenase | -1.03 | 0.030 |
| Nucb2 | nucleobindin 2 | -1.06 | 0.010 |
| Nudt19 | nudix (nucleoside diphosphate linked moiety X)-type motif 19 | 1.07 | 0.026 |
| Olfml2b | olfactomedin-like 2B | -1.13 | 0.002 |
| Osbpl10 | oxysterol binding protein-like 10 | -1.13 | 0.010 |
| Oxgr1 | oxoglutarate (alpha-ketoglutarate) receptor 1 | 1.07 | 0.013 |
| P2rx4 | purinergic receptor P2X, ligand-gated ion channel 4 | 1.03 | 0.003 |
| Pak1 | p21 protein (Cdc42/Rac)-activated kinase 1 | 1.13 | 0.028 |
| Pak6 | p21 protein (Cdc42/Rac)-activated kinase 6 | -1.11 | 0.004 |
| Pde3a | phosphodiesterase 3A, cGMP inhibited | 1.07 | 0.034 |
| Pde6d | phosphodiesterase 6D, cGMP-specific, rod, delta | -1.05 | 0.001 |
| Pfas | phosphoribosylformylglycinamidine synthase (FGAR amidotransferase) | 1.07 | 0.003 |
| Phf20 | PHD finger protein 20 | 1.05 | 0.032 |
| Pigx | phosphatidylinositol glycan anchor biosynthesis, class X | -1.05 | <0.001 |
| Pik3c2g | phosphatidylinositol 3-kinase, C2 domain containing, gamma polypeptide | -1.07 | 0.029 |
| Pla2g5 | phospholipase A2, group V | 1.11 | <0.001 |
| Plac9 | placenta specific 9 | 1.18 | <0.001 |
| Plat | plasminogen activator, tissue | 1.05 | 0.044 |
| Pld1 | phospholipase D1 | -1.11 | 0.005 |
| Plekhb1 | pleckstrin homology domain containing, family B (evectins) member 1 | 1.28 | <0.001 |
| Plxdc1 | plexin domain containing 1 | -1.14 | 0.003 |
| Polr1b | polymerase (RNA) I polypeptide B | -1.07 | 0.050 |
| Ppm1l | protein phosphatase 1 (formerly 2C)-like | -1.05 | 0.025 |
| Prkcq | protein kinase C, theta | 1.03 | 0.041 |
| Procr | protein C receptor, endothelial | 1.09 | 0.014 |
| Prom2 | prominin 2 | -1.07 | 0.024 |
| Psmb3 | proteasome (prosome, macropain) subunit, beta type 3 | -1.03 | 0.017 |
| Pter | phosphotriesterase related | -1.03 | 0.008 |
| Pxk | PX domain containing serine/threonine kinase | 1.03 | 0.002 |
| Rbfa | RIKEN cDNA 1110032A13 gene | 1.04 | 0.003 |
| Rbp7 | retinol binding protein 7, cellular | 1.25 | 0.044 |
| Rgs5 | regulator of G-protein signaling 5 | 1.06 | 0.025 |
| Rhbg | Rhesus blood group-associated B glycoprotein | 1.08 | 0.003 |
| Rhobtb2 | Rho-related BTB domain containing 2 | 1.06 | 0.029 |
| Rnf13 | ring finger protein 13 | 1.06 | <0.001 |
| Rnf187 | ring finger protein 187 | -1.05 | 0.002 |
| Rpap2 | RNA polymerase II associated protein 2 | 1.05 | 0.020 |
| Rpgrip1l | Rpgrip1-like | -1.05 | 0.019 |
| Rpl15 | ribosomal protein L15 | -1.02 | 0.020 |
| Rpl26l1 | predicted pseudogene 10136 | -1.05 | 0.003 |
| Rpl35a | ribosomal protein L35A | -1.03 | 0.037 |
| Rps13 | ribosomal protein S13 | 1.03 | 0.023 |
| Rsph3a | radial spoke 3A homolog (Chlamydomonas) | 1.06 | 0.015 |
| Rtf1 | Rtf1, Paf1/RNA polymerase II complex component, homolog (S. cerevisiae) | 1.05 | <0.001 |
| Scn2b | sodium channel, voltage-gated, type II, beta | 1.12 | 0.007 |
| Scnn1b | sodium channel, nonvoltage-gated 1 beta | 1.05 | 0.009 |
| Scrg1 | scrapie responsive gene 1 | 1.63 | 0.001 |
| Sectm1 | secreted and transmembrane 1A | -1.41 | <0.001 |
| Sectm1b | secreted and transmembrane 1B | -1.16 | 0.002 |
| Sema5a | sema domain, seven thrombospondin repeats (type 1 and type 1-like), transmembrane domain (TM) and short cytoplasmic domain, (semaphorin) 5A | 1.05 | 0.005 |
| Serinc3 | serine incorporator 3 | 1.03 | <0.001 |
| Serpina1f | serine (or cysteine) peptidase inhibitor, clade A, member 1F | 1.07 | <0.001 |
| Serpinb8 | serine (or cysteine) peptidase inhibitor, clade B, member 8 | -1.22 | <0.001 |
| Serpinh1 | serine (or cysteine) peptidase inhibitor, clade H, member 1 | -1.08 | 0.038 |
| Sfxn1 | sideroflexin 1 | -1.02 | 0.008 |
| Shisa7 | shisa homolog 7 (Xenopus laevis) | -1.05 | 0.028 |
| Slc12a7 | solute carrier family 12, member 7 | -1.05 | 0.011 |
| Slc15a2 | solute carrier family 15 (H+/peptide transporter), member 2 | 1.06 | <0.001 |
| Slc16a5 | solute carrier family 16 (monocarboxylic acid transporters), member 5 | -1.26 | <0.001 |
| Slc25a10 | solute carrier family 25 (mitochondrial carrier, dicarboxylate transporter), member 10 | -1.06 | 0.005 |
| Slc26a1 | solute carrier family 26 (sulfate transporter), member 1 | -1.08 | 0.003 |
| Slc35e1 | solute carrier family 35, member E1 | -1.04 | 0.025 |
| Slc5a6 | solute carrier family 5 (sodium-dependent vitamin transporter), member 6 | -1.10 | 0.018 |
| Slc6a13 | solute carrier family 6 (neurotransmitter transporter, GABA), member 13 | -1.05 | 0.045 |
| Slco1a6 | solute carrier organic anion transporter family, member 1a6 | 1.08 | <0.001 |
| Slit2 | slit homolog 2 (Drosophila) | 1.06 | 0.023 |
| Slitrk6 | SLIT and NTRK-like family, member 6 | 1.32 | 0.028 |
| Snord53 | small nucleolar RNA, C/D box 53 | 1.31 | 0.001 |
| Snx31 | sorting nexin 31 | 1.11 | 0.047 |
| Sorbs3 | sorbin and SH3 domain containing 3 | 1.05 | 0.007 |
| Spink6 | serine peptidase inhibitor, Kazal type 6 | -1.88 | <0.001 |
| Spns2 | spinster homolog 2 (Drosophila) | -1.04 | 0.023 |
| Sppl2a | RIKEN cDNA 2010106G01 gene | 1.03 | 0.030 |
| Sptlc3 | serine palmitoyltransferase, long chain base subunit 3 | 1.10 | 0.004 |
| Sqrdl | sulfide quinone reductase-like (yeast) | -1.06 | <0.001 |
| St7 | suppression of tumorigenicity 7 | -1.08 | 0.001 |
| Stard10 | START domain containing 10 | -1.02 | 0.003 |
| Stx18 | syntaxin 18 | -1.03 | 0.031 |
| Stxbp5l | syntaxin binding protein 5-like | -1.07 | 0.016 |
| Sympk | symplekin | -1.03 | 0.028 |
| Tc2n | tandem C2 domains, nuclear | 1.15 | 0.006 |
| Tex15 | testis expressed gene 15 | 1.41 | <0.001 |
| Tfpi | tissue factor pathway inhibitor | 1.05 | 0.018 |
| Thbs1 | thrombospondin 1 | 1.04 | 0.026 |
| Thsd7a | thrombospondin, type I, domain containing 7A | 1.07 | 0.027 |
| Tmc4 | transmembrane channel-like gene family 4 | 1.04 | 0.004 |
| Tmco1 | transmembrane and coiled-coil domains 1 | 1.04 | <0.001 |
| Tmed5 | transmembrane emp24 protein transport domain containing 5 | 1.06 | 0.031 |
| Tmem20 | transmembrane protein 20 | 1.04 | 0.050 |
| Tmem39b | transmembrane protein 39b | 1.08 | <0.001 |
| Tmem8a | transmembrane protein 8 (five membrane-spanning domains) | -1.05 | <0.001 |
| Tomm34 | translocase of outer mitochondrial membrane 34 | -1.04 | 0.020 |
| Tox | thymocyte selection-associated high mobility group box | 1.15 | 0.023 |
| Trappc2l | trafficking protein particle complex 2-like | -1.08 | 0.001 |
| Trim13 | tripartite motif-containing 13 | -1.07 | <0.001 |
| Tspan15 | tetraspanin 15 | 1.10 | 0.004 |
| Ube2c | ubiquitin-conjugating enzyme E2C | -1.24 | 0.019 |
| Ubiad1 | UbiA prenyltransferase domain containing 1 | 1.06 | 0.016 |
| Ugt3a1 | UDP glycosyltransferases 3 family, polypeptide A1 | -1.02 | 0.013 |
| Usp53 | ubiquitin specific peptidase 53 | 1.07 | <0.001 |
| Vmn2r37 | vomeronasal 2, receptor 37 | -1.11 | 0.012 |
| Vmn2r43 | vomeronasal 2, receptor 43 | -1.14 | 0.012 |
| Vwa1 | von Willebrand factor A domain containing 1 | -1.14 | <0.001 |
| Vwa5a | von Willebrand factor A domain containing 5A | -1.03 | 0.010 |
| Zcchc14 | zinc finger, CCHC domain containing 14 | -1.05 | 0.005 |
| Zfp106 | zinc finger protein 106 | 1.05 | 0.012 |
| Zfp125 | zinc finger protein 125 | -1.67 | 0.006 |
| Zfp738 | zinc finger protein 738 | 1.09 | 0.003 |
| Znf426 | zinc finger protein 426 | -1.06 | <0.001 |
| Znf605 | zinc finger protein 605 | 1.37 | <0.001 |
| Znf830 | zinc finger protein 830 | 1.17 | 0.016 |
| Zpld1 | zona pellucida like domain containing 1 | 1.30 | <0.001 |
